# How I Manage Transplant Ineligible Patients with Myelodysplastic Neoplasms

**DOI:** 10.1007/s44228-022-00024-4

**Published:** 2022-12-27

**Authors:** Carmelo Gurnari, Zhuoer Xie, Amer M. Zeidan

**Affiliations:** 1 Translational Hematology and Oncology Research Department, Taussig Cancer Center, Cleveland Clinic, Cleveland, OH USA; 2 Department of Biomedicine and Prevention, University of Rome Tor Vergata, Rome, Italy; 3 Department of Hematology, H. Lee Moffitt Cancer Center, Tampa, FL USA; 4 Section of Hematology, Department of Medicine, Yale School of Medicine, and Yale Cancer Center, New Haven, CT USA

**Keywords:** Myelodysplastic neoplasms, Clinical management, Tailored approaches, Treatment options

## Abstract

Myelodysplastic neoplasms, formerly known as myelodysplastic syndromes (MDS), represent a group of clonal disorders characterized by a high degree of clinical and molecular heterogeneity, and an invariable tendency to progress to acute myeloid leukemia. MDS typically present in the elderly with cytopenias of different degrees and bone marrow dysplasia, the hallmarks of the disease. Allogeneic hematopoietic stem cell transplant is the sole curative approach to date. Nonetheless, given the disease’s demographics, only a minority of patients can benefit from this procedure. Currently used prognostic schemes such as the Revised International Prognostic Scoring System (R-IPSS), and most recently the molecular IPSS (IPSS-M), guide clinical management by dividing MDS into two big categories: lower- and higher-risk cases, based on a cut-off score of 3.5. The main clinical problem of the lower-risk group is represented by the management of cytopenias, whereas the prevention of secondary leukemia progression is the goal for the latter. Herein, we discuss the non-transplant treatment of MDS, focusing on current practice and available therapeutic options, while also presenting new investigational agents potentially entering the MDS therapeutic arsenal in the near future.

## Introduction

Myelodysplastic neoplasms (MDS), previously known as myelodysplastic syndromes, are a heterogeneous group of clonal disorders of the hematopoietic stem cell (HSC), presenting with variable degrees of anemia, thrombocytopenia and neutropenia [1]. The natural history of MDS is characterized by an invariable tendency to secondary leukemia progression, specifically in cases with proliferation of bone marrow (BM) blasts at disease onset [2]

MDS is a disease of the elderly, with a median age at diagnosis of approximately 70 years. The disease’s incidence ranges from 4 per 100,000 in the general population to 25–40 per 100,000 in people aged > 65 years [2, 3]. The rare instances in younger patients (< 50 years of age) should prompt genetic testing for inherited conditions, in order to adequately address familial counseling and potential donor choice for allogeneic hematopoietic stem cell (HSCT) purposes [4–7]. The latter represents the only curative treatment to date, but its wide application is limited by the disease demographics, and only eligible patients can benefit from this procedure [8, 9]. Late-onset and incomplete penetrance, and inherited predisposition traits may be present also in older MDS cases, with *DDX41*-mutants as prototypical examples [10, 11].

**Fig. 1:**
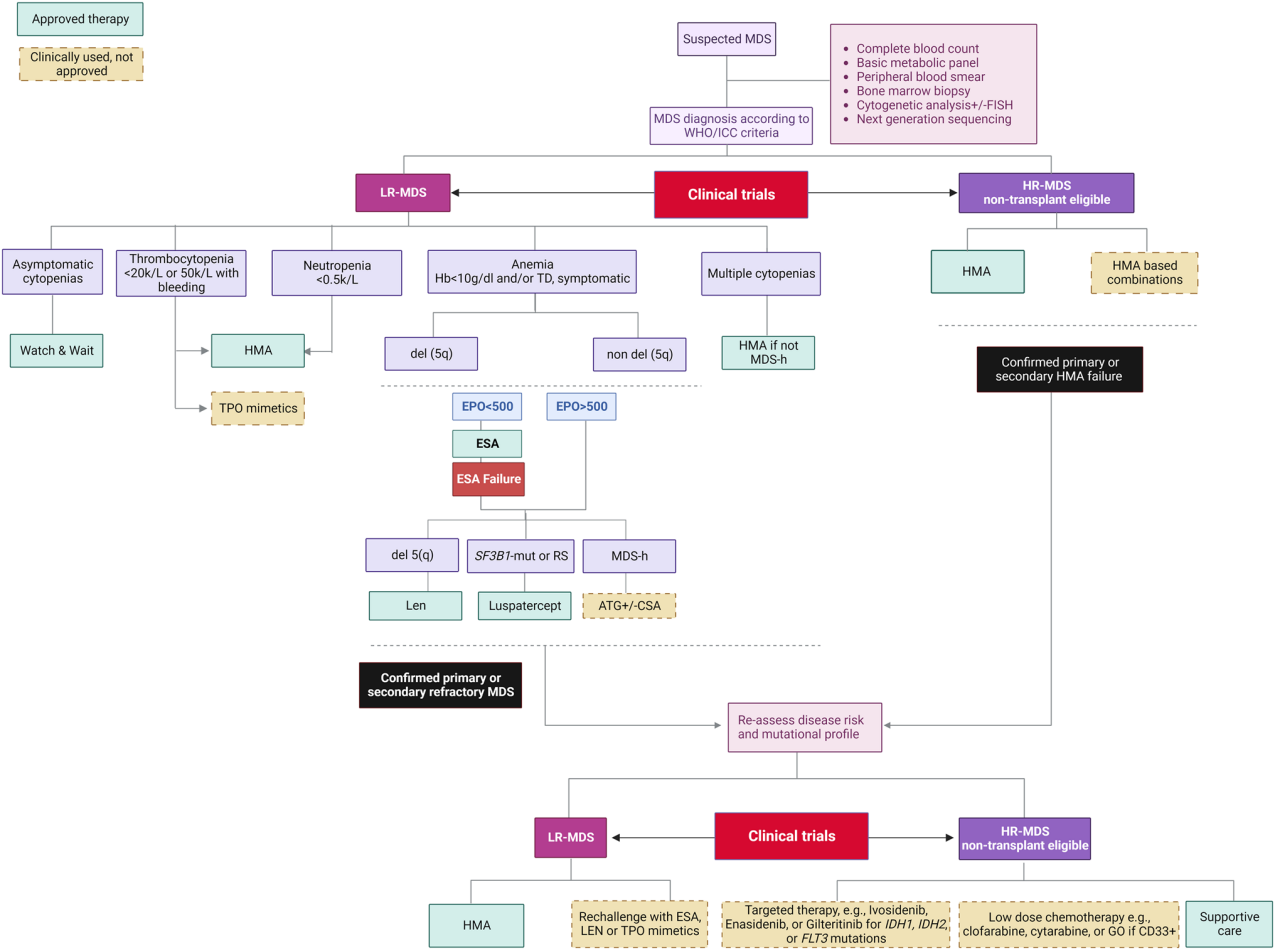
Management of non-transplant eligible patients with MDS according to the 2022 NCCN and 2021 ESMO Guidelines [3, 34]. The diagram displays the initial diagnostic evaluation and treatment algorithm for MDS according to the International Prognostic Scoring System (R-IPSS) at onset. Abbreviations: *EPO* Erythropoietin, *ESA* Erythropoiesis-stimulating agents, *HMA* hypomethylating agents, *HR* higher-risk, *Len* lenalidomide, *LR* lower-risk, *MDS* myelodysplastic neoplasms, *TPO* thrombopoietin receptor agonist

Clinically, the degree of cytopenias spans from mild to severe, with up to one-third of cases being already transfusion-dependent at disease onset [2]. Therefore, signs and symptoms are related to the type and severity of peripheral blood (PB) cytopenias, with anemia being the most common form. Differential diagnoses include other hematological conditions characterized by bone marrow failure/insufficiency such as aplastic anemia, paroxysmal nocturnal hemoglobinuria (PNH), pure red cell aplasia, as well as nutritional, endocrine and metabolic alterations [12–15]. Figure 1 (upper part) summarizes the initial laboratory assessment needed for the work-up of a patient with suspicion of MDS.

**Fig. 2:**
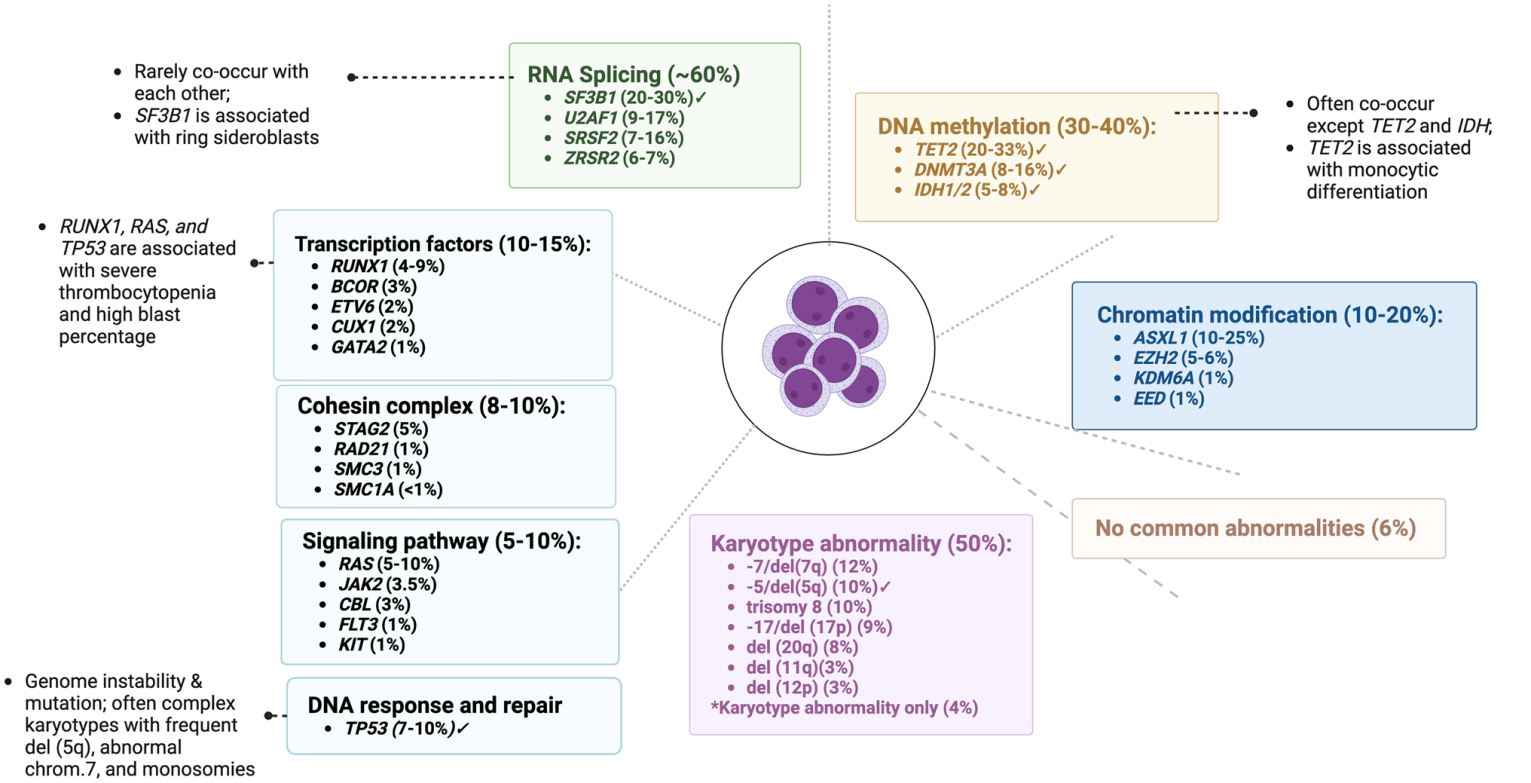
MDS genomic landscape. The figure highlights gene mutations found in myelodysplastic neoplasms (MDS) and their frequencies. √ indicates the available targeted therapeutics

In the last decade, the introduction of next-generation sequencing (NGS) has unveiled a plethora of gene mutations associated with MDS pathogenesis, thereby inaugurating a new molecular era (Fig. 2) [16, 17]. Paralleling the observed clinical heterogeneity, these studies uncovered that MDS patients also harbor a variety of genomic alterations, some of them identifying specific prognostic sub-entities [18]. The association between specific molecular lesions and peculiar MDS subtypes underpins the updates included in the most recent edition of the World Health Organization (WHO) classification (Table 1). Besides confirming already known molecularly-defined sub-entities such as MDS with low blast counts and isolated del(5q), the first genomic alteration introduced in MDS classification [19], the 5th WHO edition now includes two additional subtypes with defining genetic abnormalities: MDS with low blast counts and *SF3B1* mutation, and MDS with biallelic *TP53* inactivation [20]. Evaluation of BM blasts, cellularity and fibrosis and exclusion of cytogenetic and/or molecular features configuring one of the above-mentioned MDS subtypes are consequential for the definition of the rest of the cases (so called, morphologically defined) [20]. Of note, a blast threshold of ≥ 20% is still retained for the definition of acute myeloid leukemia (AML), whereas a 10% cut-off serves for the identification of the new sub-entity of MDS/AML, according to the International Consensus Classification of Myeloid Neoplasms and Acute Leukemia (ICC) [21]. The latter still includes a differentiation of MDS according to the absence/presence of single/multilineage dysplasia in the low blast counts (< 5% BM and < 2% PB) category defined as not otherwise specified (NOS), reminiscent of the previous WHO classification [22] (Table 1).

**Table 1:** Myelodysplastic neoplasms/Myelodysplastic syndrome classification according to the 5th WHO 2022 edition, adapted from [20]

Disease subtype	Blasts	Cytogenetics
*MDS with defining genetic abnormalities*		
MDS with biallelic *TP53* inactivation^a^	< 20% BM and PB	Usually complex
MDS with low blasts and isolated del(5q)	< 5% BM and < 2% PB	Isolated del(5) or plus 1 additional abnormality other than − 7/del(7q)
MDS with low blasts and *SF3B1* mutation^b^		Absence of del(5q), − 7, or CK
*MDS, morphologically defined*		
MDS with low blasts (MDS-LB)	< 5% BM and < 2% PB	
MDS, hypoplastic (MDS-h)		
MDS with increased blasts 1 (MDS-IB1)	5–9% BM or 2–4% PB	
MDS with increased blasts 2 (MDS-IB2)^c^	10–19% BM or 5–19% PB or Auer rods	
MDS with increased blasts and fibrosis (MDS-f)	5–19% BM; 2–19% PB	

^a^Defined as > 2 *TP53* mutations or 1 mutation plus copy number loss or copy-neutral loss-of-heterozygosity

^b^Requires *SF3B1* mutation

^c^A blast threshold of 10% serves for the identification of the new sub-entity of MDS/AML according to the ICC [21] which also still retain a differentiation according to the absence/presence of single/multilineage dysplasia in the low blasts (< 5% BM and < 2% PB) category defined as not otherwise specified (NOS)

### Risk-Assessment

Once the diagnosis of MDS is established and the patient is classified according to the current diagnostic schemes, the next step is to assess the risk of AML progression, paramount for treatment decisions. To this end, several prognostic tools have been proposed through the years, chiefly relying on complete blood count (CBC) parameters, BM blasts and cytogenetic alterations (Table 2) [23–25]. The existing standard is represented by the Revised International Prognostic Scoring System (R-IPSS), which accounts for the severity and number of cytopenias, percentage of BM blasts, and karyotypic abnormalities, allocating patients into five risk categories of diverse prognosis (with a median survival spanning from 9 years in Very low to less than 8 months in Very high risk groups) [26]. Generally, patients are also classified into lower (≤ 3.5) and higher-risk (> 3.5) based on the assigned R-IPSS score, identifying two subgroups of MDS with different therapeutic needs [27].

**Table 2:** Comparison of existing prognostication models in MDS

Model variables	Cytogenetic risk groups	BM blasts %	Cytopenia	Molecular information	Patient’s factor	C-index
Model names						
*Models without molecular information*						
IPSS [23] 1997	Y	Y	Number of cytopenias		X	0.65
IPSS-R [26] 2002	Y	Y	Degree of cytopenias		X	0.67
WPSS [25] 2007	Y	WHO category	Transfusion requirement		X	0.65
MDACC [24] 2008	Y	Y	Prior transfusion; Degree of cytopenias		Age; Performance status	0.65
*Models with molecular information*						
Haferlach et al. [18] 2014	Y	Y	Degree of cytopenias (PLT, Hb)	*ASXL1, CBL, ETV6, EZH2, KRAS, LAMB4, NCOR2, NF1, NPM1, NRAS, PRPF8, RUNX1, TET2, TP53*	Age Gender	NA
Nazha et al. [28] 2016	Y	WHO category	According to the combination models	*TP53, EZH2*, and *SF3B1*	Age	0.71
Nazha et al. [29] 2021	Y	Y	Degree of cytopenias (PLT, Hb)	*RUNX1, RAD21, SRSF2, SF3B1, STAG2, ASXL1, TP53*	Age	0.74
Bersanelli et al. [17] 2021	Y	WHO category	Number of cytopenias	47 molecular variables	Age Gender	0.75
IPSS-M [32] 2022	Y	Y	Degree of cytopenias (PLT, Hb)	*−* Binary (Yes/No): *ASXL1, CBL, DNMT3A, ETV6, EZH2, FLT3, IDH2, KRAS, MLLPTD, NPM1, NRAS, RUNX1, SRSF2,* and *U2AF1* *−* *TP53 allelic status* *−* *SF3B1*^*5q*^*, SF3B1*^*α*^*, SF3B1 *^*β*^ *−* Number of additional mutations in *BCOR, BCORL1, CEBPA, ETNK1, GATA2, GNB1, IDH1, NF1, PHF6, PPM1D, PRPF8, PTPN11, SETBP1, STAG2,* and *WT1*	X	0.75

Adapted from [16]. Y: yes; X: no; BM: bone marrow; PLT: platelets; Hb: hemoglobin; NA: not available; *SF3B1*^*5q*^*: SF3B1* plus isolated del(5q); *SF3B1*^*β*^*: SF3B1 plus BCOR, BCORL1, NRAS, RUNX1, SRSF2, or STAG2; SF3B1*^*α*^ any other *SF3B1*-mutant; *TP53* allelic status is defined according to [105]. C-indexes refer to overall survival prediction

The recent advances in molecular biology have made the incorporation of genomic information into MDS prognostication a paramount task, eagerly pursed by the entire community [28, 29]. Artificial intelligence (AI) and machine learning algorithms have also demonstrated converging pathobiological routes by unveiling latent commonalities, but have also highlighted patterns unique to specific MDS clusters with prognostic implications [17, 29, 30]. Besides obvious potentialities, current pitfalls of such approaches lie in the statistical power of the sample size needed in case of rare mutational events, the consideration of specific mutational characteristics (e.g., variant allelic frequency-VAF, type of mutations), and the inherent ‘black-box’ nature of the AI methods [31].

In an attempt to address the unmet need of incorporating the abundance of genomic information into MDS prognostication, the International Working Group for the Prognosis of MDS has recently developed the Molecular International Prognostic Scoring System (IPSS-M) [32]. By combining genomic profiling, cytogenetic and hematologic parameters, the IPSS-M is able to better predict survival and leukemic progression in patients with MDS as compared to its prior R-IPSS version, as demonstrated by the increased C-index reported in the study (0.75 versus 0.67, respectively; Table 2). This notwithstanding, validation of this new tool in real-life setting is required prior to its routine use in clinical practice for treatment decisions and patient’s allocation for clinical trials.

### Clinical Management

#### Treatment of Lower-Risk MDS

As mentioned above, the clinical management of MDS is based on the initial risk-assessment according to IPSS-R scores. Taking into account the more favorable natural history of lower-risk MDS, the treatment strategy in such cases mainly focuses on improvement of cytopenias and related symptoms with MDS-directed approaches or supportive measures (Fig. 1, left side). Nevertheless, in some instances of asymptomatic lower-risk patients with mild-to-moderate cytopenias, a “watch and wait” strategy can be advisable [33]. In this scenario, the better understanding of the disease pathobiology has shed light on the potential implications of the use of NGS, and future studies exploring early interventions following genomic profiling are warranted [16].

Treatment choices depend on the clinical presentations. Anemia is one of the most typical features at diagnosis in lower-risk disease. In these cases, the first-line option is represented by erythropoiesis-stimulating agents (ESA) in both European and USA expert recommendation panels [3, 34]. Randomized clinical trials showed that ESA at a weekly EPO dose of 30–80,000 units yield an overall response rate (ORR) of up to 40–50% within 8–12 weeks from treatment start, and with a median duration of response of 15–24 months [35–37]. ORR to ESA and achievement of transfusion-independence are strictly dependent on baseline EPO in an inverse fashion, with levels below 200 IU/L associated with the best outcomes [38, 39]. Conversely, patients with EPO levels above 500 IU/L have reduced likelihood of erythroid response and must be offered alternative treatment options or clinical trial enrollment, whenever possible [40]. When EPO levels are increased, the response mechanisms to anemia may have already reached a supraphysiologic threshold of stimulation, as demonstrated by the observed expansion of erythropoiesis in BM evaluation at diagnosis in these cases [41]. Other factors influencing response to ESA include the concomitant administration of Granulocyte-Colony Stimulating Factor (G-CSF), the IPSS-R score and burden of prior transfusion requirements [36, 42].

Approximately 10–15% of MDS carry interstitial deletions of the long arm of chromosome 5, namely del(5q). [43] MDS with del(5q) exhibit an exquisite sensitivity to lenalidomide, which represents the first targeted treatment for MDS [19]. Clinical trials have shown erythroid responses in 70–80% of cases, with achievement of transfusion-independence for up to a median of 2.5 years [44, 45]. Age at onset, neutrophil counts and lactate dehydrogenase levels, as well as the achievement of erythroid and cytogenetic responses have been identified as predictive factors for overall and progression-free survival [46]. Lenalidomide is given orally at a dose of 10 mg/daily for 21 days in 28-days cycles and, particularly during the first cycles, may cause neutropenia and thrombocytopenia. These treatment-related cytopenias have been linked to the cytotoxic suppression of the actual del(5q) clone, thereby leading to higher likelihood of response [47]. Data on long-term outcomes showed a safety signal of the drug with reassurance as to the worrisome rumors concerning secondary neoplasms and leukemia progression. The observation of long-term remissions following treatment stop led to the current interest in exploring treatment-free remission strategies, paralleling the experience in other hematologic diseases such as chronic myeloid leukemia [46, 48, 49]. This notwithstanding, up to 20% of MDS with del(5q) harbor *TP53* mutations, which have been associated to an increased risk of secondary leukemia progression, albeit without preventing initial erythroid responses [50, 51]. Therefore, NGS evaluation is recommended to better identify cases at higher risk of AML progression, and may have a role in guiding treatment-free remission strategies, as previous studies have shown that some malignant HSC carrying del(5q) may still persist following the achievement of complete remission (CR) [52].

MDS with ring sideroblasts (RS), usually associated with *SF3B1* mutations, typically present with anemia that can benefit from luspatercept, an activin receptor ligand trap inhibitor of the transforming growth factor (TGF)-β pathway (Fig. 2). Initially tested across all lower-risk MDS in an open-label phase 2 trial, luspatercept (given at a dose of 1–1.75 mg/kg every 21 days) has shown higher erythroid response rates (63%) and transfusion-independence (38%) in MDS with RS, *SF3B1* and spliceosome mutations refractory to ESA treatment [53]. The randomized, double-blind, placebo-controlled MEDALIST trial enrolled 229 ESA-refractory MDS patients with RS confirming these initial findings and the safety signal of the drug [54]. Of note is that a recent analysis of the secondary endpoints of the study showed that, besides improvement in hemoglobin, also neutrophils and platelets incremented upon luspatercept treatment [55]. Furthermore, long-term follow up data of the MEDALIST trial indicated the substantial benefit of the investigational arm with approximately 25% of patients still under treatment at more than 2 years from enrollment [56]. The phase-3 COMMANDS trial is currently comparing the efficacy and safety of luspatercept versus epoetin alfa in ESA-naïve lower-risk MDS patients regardless of the presence of RS (NCT03682536) [57].

A selected group of younger (< 60 years) lower-risk MDS cases with hypocellular marrows (defined in Table 1), multiple cytopenias, normal cytogenetics (or trisomy 8) and autoimmune-like features (small PNH clones, *STAT3*-mutant T-cell clones) can be suitable for immunosuppressive therapy with anti-thymocyte globulin (ATG) alone or more often in combination with cyclosporine A [58]. Responses are seen in up to 30–40% of cases with a median duration of 1.5 years, and seem to be associated with the presence of HLA-DR15 genotype and preferential use of horse ATG [59].

In the 30% of lower-risk MDS patients presenting with thrombocytopenia, high-dose androgens can improve platelet counts, but this effect is generally transient [3]. While not yet approved for use in the MDS setting, recent trials have explored the use of thrombopoietin-(TPO) receptor agonists (TPO-RA or TPO mimetics) [60]. In a study enrolling 250 lower-risk MDS patients randomized 2:1 to receive romiplostim or placebo weekly for a total of 58 weeks, the investigational arm achieved higher platelet counts with decreased risk of clinically significant bleeding events (relative risk, 0.92) and platelet transfusions (relative risk, 0.77), as compared to the placebo arm [60]. Despite initial concerns on increased rate of AML progression, a recent update on long-term follow-up data of this study reassured on the safety of romiplostim, showing similar progression rates between the two arms [61]. In another single-blind, randomized, phase-2 superiority trial, 90 patients were assigned 2:1 to receive eltrombopag (50–300 mg) or placebo for at least 24 weeks [62]. Similar to romiplostim, also eltrombopag was able to increase platelet counts (47% versus 3%, *p* = 0.0017) and reduce the rate of bleeding episodes (14% versus 42%, *p* = 0.0025) when compared to the placebo.

As anemia is one of the main clinical manifestations in lower-risk diseases, patients oftentimes receive red blood cell (RBC) transfusions as a supportive care measure. In such a context, the role of iron chelation therapy (ICT) is yet not fully elucidated [3, 63]. Studies have shown that ICT may potentially improve overall and progression-free survival in transfusion-dependent lower-risk MDS. In a meta-analysis collating data from nine observational studies [64], overall ICT was associated with lower risk of mortality (HR 0.42; 95% CI 0.28–0.62; *p* < 0.01) but not with decreased rates of AML progression. In the recent multicenter, double-blind, placebo-controlled trial TELESTO [65], 225 MDS patients with iron overload (serum ferritin levels > 2247 pmol/L) were randomly assigned (2:1) to deferasirox (10–40 mg/kg per day orally) or placebo. The study showed a better event-free survival in the deferasirox arm and a clinically manageable safety profile of the drug, thereby concluding in favor of using ICT in such a setting.

#### Treatment of Higher-Risk MDS

Besides HSCT, which is outside the scope of this review focusing on management of non-transplant eligible patients, hypomethylating agents (HMA) are the backbone treatment option for higher-risk cases (Fig. 1, right side) [3, 34]. Azacitidine (AZA) is usually administered subcutaneously at a dose of 75 mg/m^2^ daily for 7 days of a 28-day cycle, whereas decitabine (DEC) is given as an intravenous formulation at a dose of 20 mg/m^2^ daily for 5 days of a 28-day cycle [2]. In the open-label, phase-3 AZA-001 trial [66], 358 patients with higher-risk MDS were randomly assigned in a 1:1 fashion to receive AZA or conventional care (low-dose cytarabine, intensive chemotherapy or best supportive care). The results of the study showed a benefit of AZA in terms of ORR (29% versus 12%) and overall survival, with 51% versus 26% of patients alive at 2 years, as compared to the control arm. However, the reported survival advantage has been modest in subsequent trials, and many combinations with different drugs including checkpoint inhibitors (e.g., durvalumab) or targeted therapies (e.g., glasdegib, histone deacetylase inhibitors) have been tried without incremental improvements of outcomes, likely due to additional toxicities [67–69]. DEC has also been shown to increase ORR rate and prolong disease-free (but not overall) survival in randomized clinical trials [70, 71]. Nevertheless, in a study on long-term outcomes of higher-risk MDS patients treated with HMA and not undergone subsequent HSCT, only 4% of cases were alive at 5 years from treatment start, regardless of the type of HMA used [72]. Therefore, participation of patients not eligible for HSCT in clinical trials is strongly recommended, especially after HMA failure [73, 74]. Of note is that no difference in rates of CR, ORR and survival has been shown between the two drugs AZA and DEC, and this finding has been demonstrated not only in MDS but also in AML patients unfit for intensive chemotherapy [75, 76].

Given logistic difficulties with on-label administration schedules of AZA, various alternatives have been explored [77]. For instance, a 5-2-2 type of regimen (Monday-Friday with weekend off and then Monday-Tuesday) is widely used by many centers (85% of 105 US centers according to a registry study [78]) to avoid the issue of weekend infusions, but no clinical randomized trial supports its equivalence with the 7–0 approved schedule [79]. Alternative regimens aside, a mainstay for a successful treatment with AZA is its continuation with a correct timing for at least the initial 4–6 cycles, in order to avoid rapid loss of response upon withdrawal due to related toxicities [80]. An alternative approach to HMA, albeit inferior to AZA, is low-dose cytarabine (LDAC) at a dose of 20 mg/m^2^/day for 10–14 days every 4 weeks [66].

Apart from schedules and therapy adherence, the response to HMA varies according to other baseline patient characteristics, cytogenetics and molecular alterations [16, 81]. For instance, male gender and burden of comorbidities have been linked to inferior response rates [82–84]. Chromosome 7 abnormalities, 17p deletion (and thus *TP53* disruption), and chromosomal translocations are also predictors of poor response and outcomes following AZA therapy [85, 86]. Conversely, an increase in platelet counts after the first cycles has been identified as a favorable prognostic factor for treatment response, and mechanistically linked to transcriptomic changes in factors implicated in late megakaryopoiesis [85, 87].

**Fig. 3:**
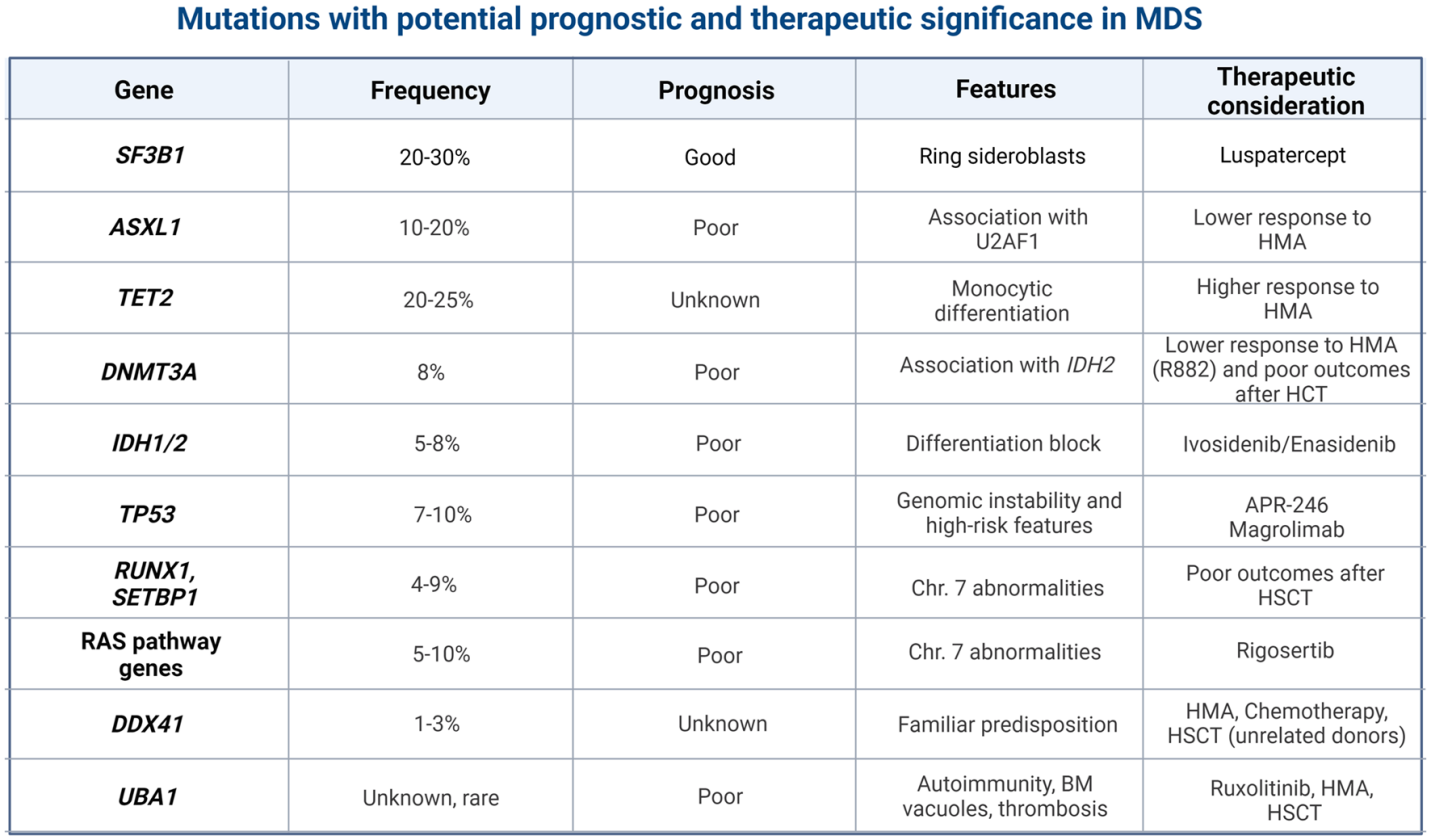
Mutations with potential prognostic and therapeutic significance in MDS. The figure highlights potential gene mutations found in patients with MDS associated with prognostic significance or sensitivity to specific treatments

While *TET2*- (with controversial results) and *DDX41*-mutants seem to have a higher rate of HMA response, MDS patients harboring *ASXL1* and specific *DNMT3A* variants (e.g., R882H) tend to have dismal outcomes (Fig. 3) [88–90]. Another instance where HMA seems to be promising is the clinical dyad of MDS and VEXAS syndrome, a new hematoinflammatory disorder characterized by the concomitant presence of BM vacuoles, autoinflammatory symptoms, macrocytic anemia, and MDS in up to 60% of cases [91, 92]. In this context, treatment with AZA achieved CR not only of the underlying MDS but also of the corollary autoinflammatory manifestations, with disappearance of the *UBA1*-mutant pathogenic clone in some cases [93, 94].

No established guidelines exist to inform on treatment choices after HMA failure in patients not eligible for transplant, and clinical trial enrollment is recommended in such cases. However, off-label options may be represented by molecularly targeted therapies as well as molecularly-agnostic options (see below) [95].

### A Glimpse into the Future: Brief Overview of New Treatments and Investigational Agents

**Fig. 4:**
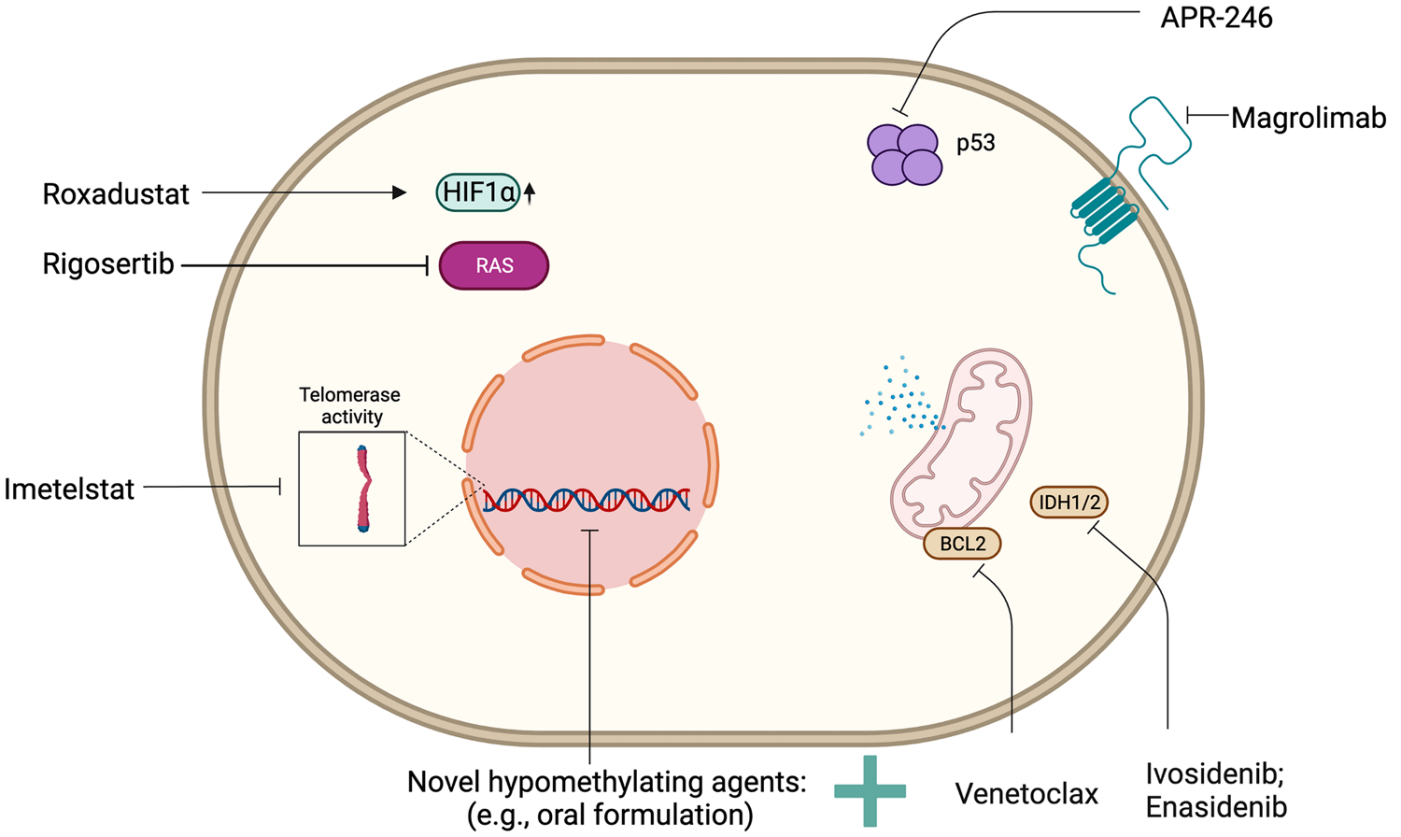
Actionable targets and investigational agents in MDS. The figure showcases actionable targets identified in MDS and currently investigated agents with potential for future use in the routine clinical management

In the last decade, the better understanding of the molecular biology of the disease has opened new therapeutic possibilities, borrowed from the experience matured in other hematological disorders. While several new drugs targeting specific pathways crucial for MDS are currently investigated, we will focus herein on some agents potentially entering routine clinical practice in the next few years (Fig. 4) [96].

Novel oral formulations of HMA (both AZA and DEC) are currently under evaluation alone or in combination with other drugs. The DEC and cedazuridine combination has been recently approved in the US and its benefit has been proven in a phase 3 study (ASCERTAIN) enrolling 133 patients with MDS and chronic myelomonocytic leukemia (CMML), showing an ORR of 60% [97]. Pharmacokinetic studies confirmed that the oral route of administration, potentiated with the addition of cedazuridine to prevent the inactivation of DEC in the gastrointestinal tract, led to systemic levels of the drug similar to those obtained with the injectable form [98, 99]. An oral AZA formulation (CC-486) has been tested in 216 MDS RBC transfusion-dependent patients in a phase 3 randomized (1:1), placebo-controlled trial [100]. While not improving survival, CC-486 significantly ameliorated the rate of transfusion-independence (31% versus 11%) when compared to placebo. Gastrointestinal and hematological toxicity were among the most common adverse events.

Preclinical studies focusing on mechanisms of AZA resistance identified the anti-apoptotic BCL-2 family members as potent sensitizing targets [101]. Therefore, given the very promising results obtained in the AML setting [102], efforts to explore the combination of AZA with venetoclax, an oral BCL2 inhibitor, are undergoing in both treatment-naïve (NCT02942290; NCT04401748) and relapse/refractory higher-risk MDS (NCT02966782). Preliminary data showed ORR as high as 84%, with a median time to CR of 2.6 months achieved across all spectra of accompanying myeloid driver mutations, including *TP53* [103, 104]. The most frequent adverse events were hematological toxicities, with 45% of cases developing febrile neutropenia.

*TP53* alterations are found in up to 20% of MDS and identify a difficult-to-treat population, because of high-risk features such as complex karyotype and increased risk of AML progression [105]. In such a setting, two agents showed promising results. The first is APR-246, a small molecule able to restore p53 functions, which has been tested in two independent phase 2 trials in combination with AZA and induced an impressive ORR of 73%, with approximately half of patients obtaining CR [106, 107]. While confirming the higher CR rate in the investigational arm, the ongoing phase 3 study (NCT03745716) did not reach the primary endpoint, but the final results are yet to be published [16]. The second drug with potential clinical activity in *TP53*-mutant MDS is magrolimab, a first-in-class anti-CD47 antibody, which synergizes with AZA and induce “eat me” signals on leukemic stem cells by restoring macrophage-mediated phagocytosis [108]. A Phase 3, placebo-controlled trial of magrolimab in combination with AZA (ENHANCE; NCT04313881) is ongoing, and preliminary data showed CR of 40% in *TP53*-mutants higher-risk MDS [109, 110].

In the past few years, targeted anti-IDH inhibitors have shown encouraging results in AML, and thereby are now investigated in the MDS setting. Albeit at a lower frequency (5–8%), *IDH1* and *IDH2* mutations can be also found in patients with MDS, constituting amenable targets for ivosidenib and enasidenib, respectively. The use of enasidenib showed an ORR of 74% for the combination with AZA, and of up to 50% when used as a single agent in HMA-treated cases [111, 112]. Similar results have been obtained for ivosidenib alone or in combination with AZA [113]. Both anti-IDH inhibitors are currently considered off-label in higher-risk MDS patients after HMA failure while being explored in clinical trials (NCT02074839; NCT03503409; NCT03744390). Of note is that the use of these agents is associated with a differentiation syndrome characterized by a clinical picture very similar to that observed during treatment with all-trans retinoic acid in acute promyelocytic leukemia [114].

Besides the higher-risk setting, several new agents are currently studied also in lower-risk cases, chiefly directed at improvement of ineffective hematopoiesis and anemia. Roxadustat is a new inhibitor of the oxygen-sensing pathway targeting the hypoxia-inducible factor and prolyl-hydroxylase. This drug, which mimics a low oxygen status, promotes erythroid differentiation by increasing EPO levels and improving iron metabolism. Borrowing on the experience of end-stage chronic kidney disease [115, 116], roxadustat is now under evaluation for the treatment of anemia of lower-risk MDS with baseline EPO levels below 400 IU/L (NCT03263091). Preliminary results showed erythroid responses with achievement of transfusions-independence in up to 38% of patients sustained for 52 weeks [117]. Imetelstat is a first-in-class inhibitor of the human telomerase. The ongoing global phase 2/3 double-blind, placebo-controlled, randomized (2:1) IMerge trial (NCT02598661) is evaluating imetelstat in patients with non-del(5q) lower-risk MDS, ESA-resistant and naïve to HMA [118]. The results of the concluded phase 2 showed a 42% transfusion-independence rate sustained for 8 weeks, and an overall hematological improvement in 68% of cases [119].

## Conclusions

MDS constitutes a highly diverse group of disorders. First-line treatment strategies aim at improvement of cytopenias in lower-risk cases, whereas the prevention of AML progression and prolongation of survival are the main goals in the higher-risk setting [120]. In cases not suitable for HSCT, second-line approaches are strictly dependent on reassessment of the risk in a dynamic fashion, given the challenges dictated by clonal evolution and potential acquisition of additional cytogenetic or molecular alterations [121, 122]. In this scenario, off-label use of AML-approved drugs and enrollment into clinical trials represent reasonable options while waiting for the final results of ongoing studies, which very soon will broaden the therapeutic possibilities of MDS patients in a more personalized, tailored fashion.

## Data Availability

Not applicable.

## Abbreviations

AI: Artificial intelligence
ATG: Antithymocyte globulin
AML: Acute myeloid leukemia
AZA: Azacitidine
BM: Bone marrow
CBC: Complete blood count
CR: Complete response
DEC: Decitabine
ESA: Erythropoiesis-stimulating agents
G-CSF: Granulocyte-Colony Stimulating Factor
HSC: Hematopoietic stem cell
HSCT: Hematopoietic stem cell transplant
ICC: International Consensus Classification of Myeloid Neoplasms and Acute Leukemia
IPSS-R: Revised International Prognostic Scoring System
IPSS-M: Molecular International Prognostic Scoring System
MDS: Myelodysplastic neoplasms
NGS: Next-generation sequencing
NOS: Not otherwise specified
ORR: Overall response rate
PB: Peripheral blood
PNH: Paroxysmal nocturnal hemoglobinuria
TPO: Thrombopoietin-receptor agonists (TPO-RA or TPO mimetics)
WHO: World Health Organization
